# Method for the simulation of blood platelet shape and its evolution during activation

**DOI:** 10.1371/journal.pcbi.1005899

**Published:** 2018-03-08

**Authors:** Alexander E. Moskalensky, Maxim A. Yurkin, Artem R. Muliukov, Alena L. Litvinenko, Vyacheslav M. Nekrasov, Andrei V. Chernyshev, Valeri P. Maltsev

**Affiliations:** 1 Novosibirsk State University, Novosibirsk, Russia; 2 Voevodsky Institute of Chemical Kinetics and Combustion SB RAS, Novosibirsk, Russia; 3 Novosibirsk State Medical University, Novosibirsk, Russia; University of Pennsylvania, UNITED STATES

## Abstract

We present a simple physically based quantitative model of blood platelet shape and its evolution during agonist-induced activation. The model is based on the consideration of two major cytoskeletal elements: the marginal band of microtubules and the submembrane cortex. Mathematically, we consider the problem of minimization of surface area constrained to confine the marginal band and a certain cellular volume. For resting platelets, the marginal band appears as a peripheral ring, allowing for the analytical solution of the minimization problem. Upon activation, the marginal band coils out of plane and forms 3D convoluted structure. We show that its shape is well approximated by an overcurved circle, a mathematical concept of closed curve with constant excessive curvature. Possible mechanisms leading to such marginal band coiling are discussed, resulting in simple parametric expression for the marginal band shape during platelet activation. The excessive curvature of marginal band is a convenient state variable which tracks the progress of activation. The cell surface is determined using numerical optimization. The shapes are strictly mathematically defined by only three parameters and show good agreement with literature data. They can be utilized in simulation of platelets interaction with different physical fields, e.g. for the description of hydrodynamic and mechanical properties of platelets, leading to better understanding of platelets margination and adhesion and thrombus formation in blood flow. It would also facilitate precise characterization of platelets in clinical diagnosis, where a novel optical model is needed for the correct solution of inverse light-scattering problem.

## Author summary

Blood platelets are the second most numerous component of blood after red blood cells. Their main function is to stop bleeding upon vessel wall injury. Contact with foreign substances, normally absent inside the bloodstream, leads to platelet activation. After this, platelets adhere to the damaged surface and to each other, finally forming a mechanical plug. Activation is very fast and complex process, and is accompanied by dramatic change of cell shape. This morphological alteration may be vital for the prevention of blood loss. However, there is no complete understanding of its significance, maybe because the quantitative description of the platelet shape during activation is absent. Here, we describe the shape change based on the physical consideration of the platelet cytoskeleton. We propose the formulation of mathematical problem and its solution, which allows one to easily simulate a wide class of shapes for both resting and activated cells. These models are in a good agreement with platelet images observed in numerous experiments published elsewere. They may be used to simulate the shape change and hence the influence of activation on hydrodynamic, mechanical and optical properties of platelets. As a result, novel diagnostic and therapeuthic strategies and clinical implications may be obtained.

This is a PLoS Computational Biology Methods paper.

## Introduction

Human blood platelets are the paramount element of hemostasis and also contribute to a variety of other normal and pathological processes, including thrombosis, inflammation, and tumor development. These corpuscles attracted attention of early microscopists by the outstanding capacity to change physical properties in response to vessel wall injury or foreign substances [1]. The first step in reaction to such stimuli is platelet activation, which comprises a series of prothrombotic events, triggered by the increase of intracellular calcium [2–4]. Among them, morphological “disk-to-sphere” transformation is the earliest and the most notable effect. Dramatic shape change of platelet from discoid (resting) to rounded with pseudopodia (activated) occurs within seconds and leads to the alteration of optical, hydrodynamic, and mechanical properties of cells.

Quantitative description of the platelet shape and its change during activation is of great importance for various related fields. For instance, the platelet shape change affect the traces of light transmission aggregometry, a “gold standard” technique in clinical laboratories [5], and should be considered for the correct interpretation of test results [6,7]. Novel optical methods for single-cell morphological analysis [8–11] rely on the appropriate optical model for the solution of the inverse light-scattering problem. Correct description of platelet geometry is also vital for the simulation of blood cells motion in vessels [12–16], especially platelets margination and adhesion under flow [17,18]. An oblate spheroid is commonly used to approximate the platelet shape in optical and hydrodynamic simulations. This model accurately captures cell properties in a limited number of cases. Even if the influence of pseudopodia is not significant, which is the case for optical computations [19], the cell body itself may have a complicated shape. Electron microscopy images of platelets reveal the presence of cells lacking the axial symmetry [20]. While some of them only slightly deviate from the classic discoid shape, the others show large distortions, such as an overall bending. These curved cells have been thought of as an artifact for decades, but their existence is consistent with recent studies of platelet cytoskeleton [21]. There is an urgent need for a shape model which accounts for platelet microanatomical structures and captures a wide class of morphologies, including non-axisymmetric ones. Consideration of cytoskeletal elements underlying the platelet morphology is necessary for the understanding of mechanical properties of platelets (e.g., deformability), which is a topic of active experimental and computational research [22–24]. However, the model should be simple (controlled by a few tunable parameters) to facilitate the advancements in the above-mentioned fields.

Resting platelet possesses the peripheral ring (marginal band) of microtubules, which supports cell discoid shape [25–27]. Upon activation, it coils to form a saddle-like structure, which was recently observed and regarded as the principal mechanistic feature underlying cellular shape change [28]. However, the specific cause of this coiling may be related to different mechanisms (which we discuss in a specific section). Given this uncertainty, direct simulation of marginal band coiling seems to be impractical.

Instead, in this paper we propose to model coiled marginal band using a mathematical concept of the overcurved circle [29]. Such structures approximate a wide class of coiled rings formed in a variety of physical processes. Its geometry is controlled by a single parameter called the overcurvature, which increases from unity as the circle coils out of plane. We show a remarkable similarity of experimental images (published elsewhere) and the overcurved circles, which allows us to consider the marginal band overcurvature as a state variable of activation. Next, we model the outer cell membrane and the submembrane cortex as an elastic surface with constant mean curvature (or, equivalently, minimal surface area), which rests on the marginal-band skeleton and embraces a prescribed cell volume. The particular surface configuration is determined by the numerical optimization. The major advantage of this platelet shape model is that it is defined by only four parameters (or three up to the scale): length, overcurvature, thickness of the marginal band, and the cell volume. Small number of parameters is vital for massive simulations and especialy for inverse problems solutions. At the same time the model encompasses both classical discoid morphology (overcurvature = 1), and novel class of non-spheroidal, highly curved or concave shapes. We compare the simulation results with previously published platelets images and show that the new model constitutes a major improvement over the spheroidal model, still preserving reasonable simplicity. Finally, we discuss important biological and biophysical insights into platelets behavior and predict cell morphologies that have not yet been reported.

## Results

### Overcurvature describes the marginal band coiling during platelet activation

**Fig 1:**
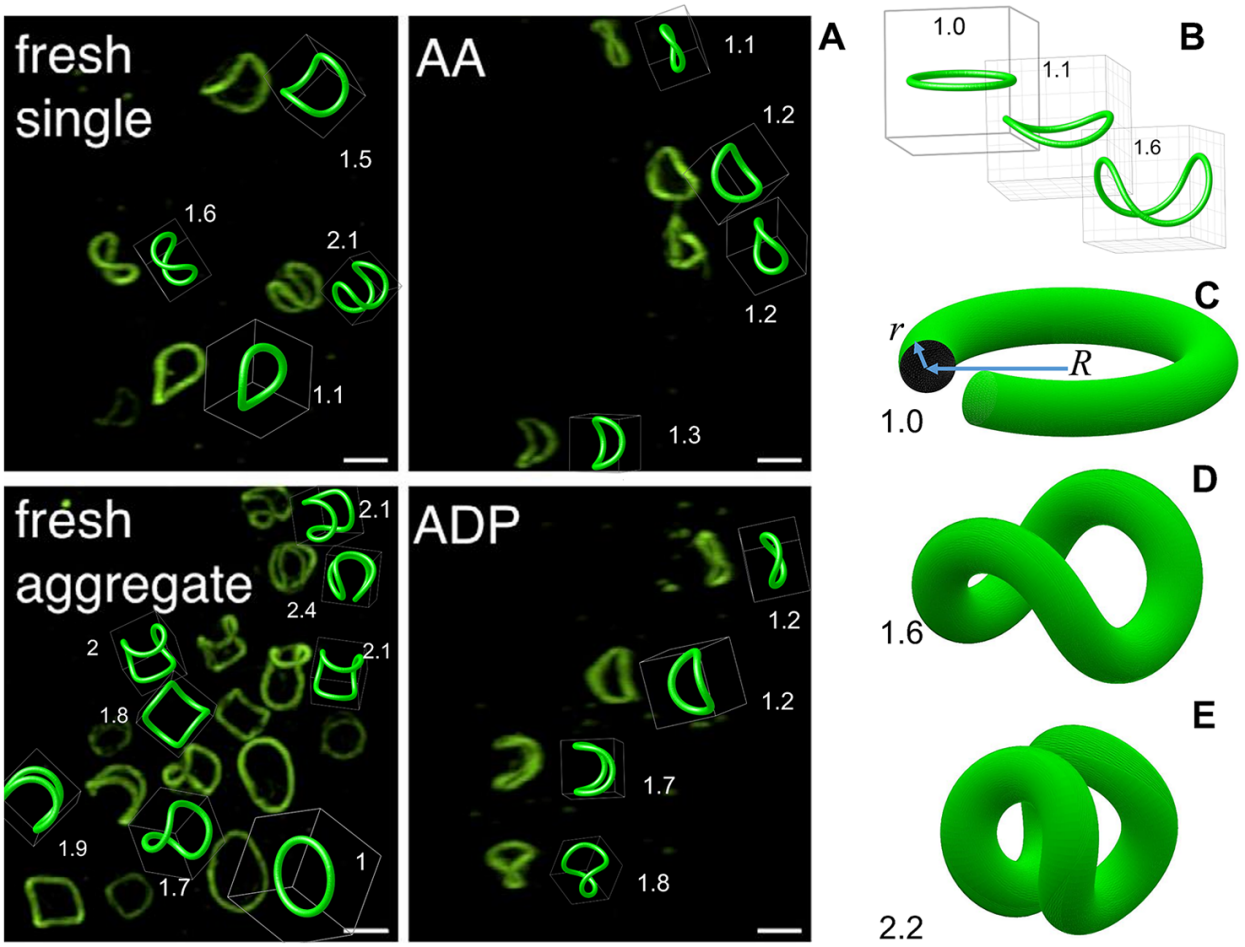
(A) One-to-one comparison of experimental images of coiled platelets marginal band (*reused with permissions from: Diagouraga et al. Journal of Cell Biology*. *204:177–185*. *DOI*: 10.1083/jcb.201306085) and manually-fitted overcurved circles. Overcurvature is listed near each plotted curve. (B) Examples of the overcurved circles. (C) Illustration of circular tube modeling the initial flat peripheral ring of microtubules. (D,E) Models of coiled marginal band in platelets with different overcurvature and non-zero thickness.

Mouthuy et al [29] showed that overcurved circles are formed in a variety of physical conditions, from curved origami to foldable tents. Surprisingly, these structures approximate marginal bands of activated platelets as well. In Fig 1A, the images of coiled marginal bands of platelets under different stimuli are reproduced from [28]. We show direct one-to-one comparison of experimental images with the overcurved circles, which manifests the remarkable agreement. An overcurvature *O*_p_ is listed near each plotted circle.

The overcurvature is the ratio between the circle’s curvature and the curvature of the same-length planar ring. As *O*_p_ increases from 1, the circle comes out of plane and adopts more and more twisted three-dimensional shape (Fig 1B). A great advantage of the overcurved circles is a simple one-parametric analytical representation. They are also invariant to the 90° rotation around the *z*-axis combined with the reflection with respect to the *xy*-plane (*D*_2d_ symmetry). Possible mechanisms leading to the formation of this particular structure in platelets are discussed in the special section, together with the mechanisms causing deviations (including the tensioned submembrane cortex).

In case of blood platelets, especially discoid cells, the thickness of marginal band can not be neglected. Further we model it as the tube of circular cross-section, whose central line forms an overcurved circle. Circular cross-section is consistent with published experimental data, although there may be reasons for compression and extension in certain directions. Two-parametric expressions for the tube surface are derived below, and several examples are shown in Fig 1C–1E. The geometry (relative shape) of platelet marginal band is therefore controlled by two parameters—the overcurvature *O*_p_ and the relative tube radius *r*/*R* (thickness); the radius of the ring in the initial (planar) state *R* is a conventional scaling parameter.

### Blood platelet outer shell as a minimal surface

First we note that the minimization of surface energy has been used for a long time to describe the biconcave shape of red blood cells (RBCs), the most studied element of blood [30,31]. Generally, this energy consists of three parts: expansion, shear, and bending [32]. The RBC surface comprises relatively weak spectrin cytoskeleton and the lipid bilayer, whose expansion or compression requires unrealistically high energy. Minimization of membrane bending energy alone at a constant area provides the shape which agrees well with experiments, at least in case of native, non-deformed RBCs [33].

By contrast, blood platelets have strong actomyosin submembrane cortex [27]. The filaments pull together nearby points on cell membrane and make it wrinkled at small scales [34]. In other words, the expansion energy is associated with the cortex, which has equilibrium surface area significantly lower than that allowed by the fixed volume. And this expansion energy is assumed to be much larger than bending energy of both the lipid bilayer (including wrinkles) and the cortex. Therefore, the description of a platelet outer shell (membrane + submembrane cortex) as a surface which tends to minimize its area is a reasonable approximation, that have been used in biophysical modeling of blood platelets [35]. We also do not account for the rigid cytoplasmic actin network, which stabilizes resting platelet shape and rapidly disassembles during activation [36]. Thus, the surface should cover the coiled marginal band and confine certain cellular volume, while minimizing the area. Such mathematical problem has not been considered elsewhere; however, one would expect that one part of surface would be attached to the marginal band and the other would be of constant mean curvature. The only difficulty is to find a boundary where the surface detaches from the marginal band. In the section “Cell surface of resting platelets”, we give a solution for axially symmetrical problem (*O*_p_ = 1) and provide the boundary conditions for the general case. We show that the contact between attached and free parts of the surface should be smooth. These considerations were used for the following simulation of platelets morphology, details of which are described in the subsequent section. Further we show attached parts of the surface in green and free surface in red.

### Platelet activation as the movement on the phase diagram

**Fig 2:**
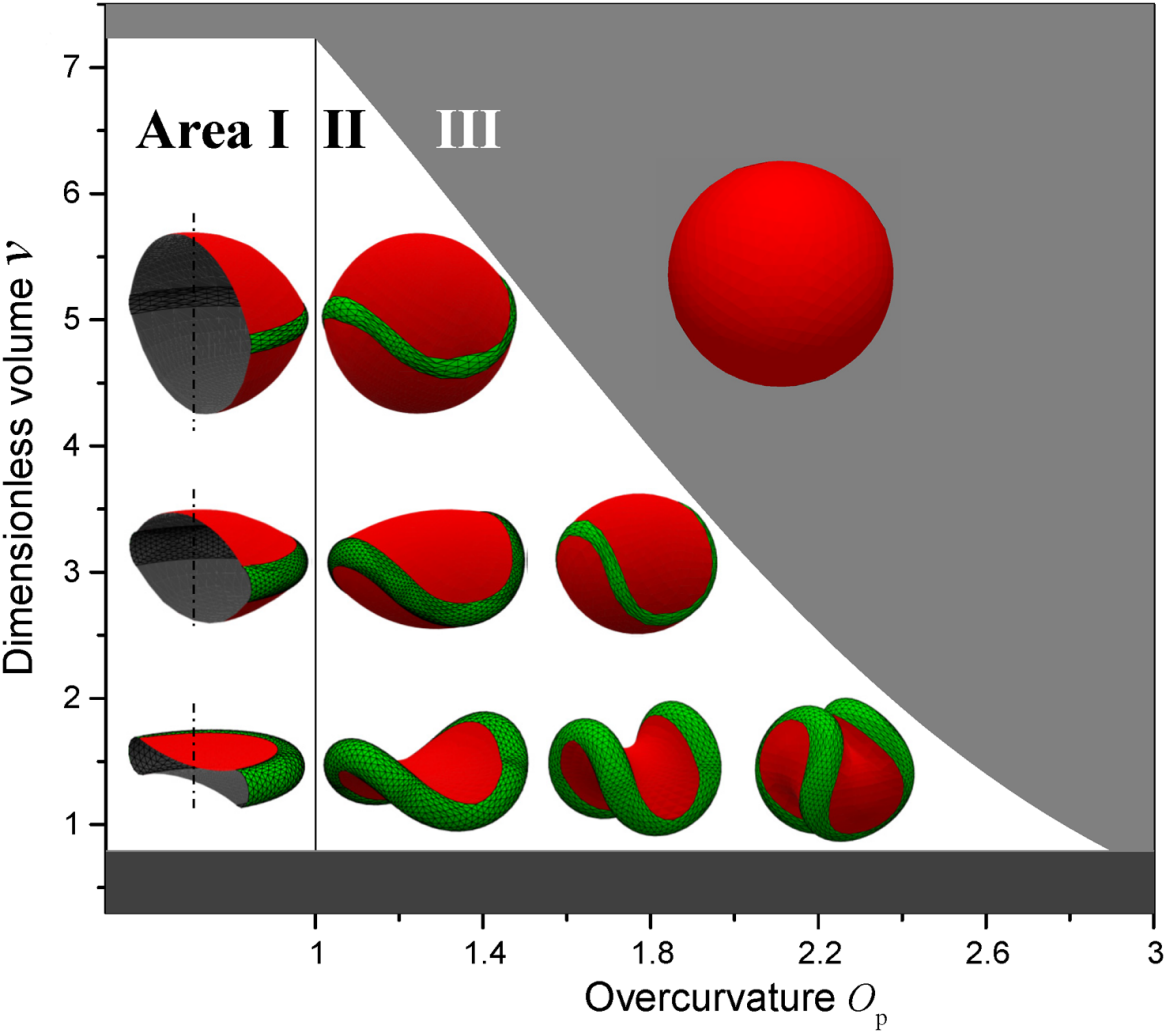
Overcurvature (*O*_p_)–Dimensionless volume (*v*) phase diagram of platelets model morphologies. Area I corresponds to resting platelets with overcurvature = 1; Area II stands for activated platelets (hypothetically reversible activation); Area III is where platelets become sphered (irreversible activation).

Marginal band in resting platelets appears as a planar ring of microtubules. In our model, it corresponds to a ring torus, characterized by *O*_p_ = 1, ring radius *R*, and the relative tube radius *r/R* < 1. The overall morphology preserves axial symmetry; in this case, the analytic solution of the surface optimization problem is possible. The resting platelet is described as the torus (marginal band) with two spherical caps covering the torus hole. Possible shapes for *R* = 1 and *r/R* = 0.2 are shown in Area I of Fig 2.

In this figure a phase diagram of possible platelets morphologies is shown in the coordinates of overcurvature *O*_p_ and the dimensioness volume *v = V*/*R*^3^, while marginal band thickness *r/R* is fixed to 0.2. Dark gray area on the bottom represents volumes less than that of torus; if the cell volume *v* is slightly above, it adopts biconcave shape, which is indeed rarely seen for platelets (see e.g. Fig. 2A in [37]). As the volume increases, caps change from inward to outward, and the cell becomes discoid and then biconvex, which corresponds to the classical platelets appearance. Further cell inflation leads to the formation of nearly spherical shapes, that are not characteristic for intact platelets, but correspond to cells after osmotic swelling [38]. Finally, the cell volume hits the boundary of area III, where it is larger than that of the sphere circumscribing the marginal band. In this case, the marginal band no longer stretches the membrane, and the cell adopts spherical shape. Hypothetically, the uneven distribution of submembrane microfilaments may cause certain regions of surface to bulge and form protrusions like pseudopodia or lamellopodia.

During activation, marginal band of platelets may alter its length and thickness, according to Diagouraga et al. [28]. For the simplicity, we assume that the relative thickness of marginal band *r/R* = 0.2 holds in this process. Activation begins with the rapid out-of-plane buckling of the marginal band, resulting in jump from area I to II in Fig 2. Area II consists of platelet shapes with *O*_p_ > 1. Note the unusual *D*_2d_ symmetry of shapes, inherited from the overcurved circles. The rightward movement on the phase diagram reflects the degree of marginal band coiling and hence the progression of activation. The cell volume may also alter upon this movement, but there is no agreement on this topic in the literature. In the region of small *v* cell surfaces preserve concave appearance, and such morphologies indeed present in experiments, see e.g. Fig. 2a in [20] and Fig. 2c in [21]. Larger cells with convex of intermediate appearance can be found in a number of recent studies, see e.g. Fig. 2A in [28] and Fig. 7A in [39]. Structures with larger values of *O*_p_, characterized by self-contact or self-intersection, have not been encountered among platelets. While self-intersection may take place for the mathematical concept of overcurved ring, it is physically impossible for the marginal band, given its non-negligible thickness. Therefore, the case of *O*_p_ > 2.2–2.4 ceases to describe platelet structure, and we do not consider such values in the present paper. However, self-intersecting structures were observed in invertebrate erythrocytes, which also have marginal band and change morphology from discoid to rounded upon blood withdrawal [40]. These cells are much larger than human platelets and have much smaller relative thickness of marginal band. Note also that the mathematical upper limit of *O*_p_ = 3 corresponds to the planar triple-folded ring, which can be coiled again to make a new overcurved circle.

The trajectories of platelets on Fig 2 during activation are not necessarily horizontal since the cell volume (and marginal band length) can vary. Nevertheless, the increase of overcurvature leads to rapid decrease of marginal band dimensions, i.e., of the volume of the circumscribing sphere. Eventually it becomes smaller than the cell volume *V*, and platelet proceeds to the zone III. The marginal band no longer stretches the membrane, which may lead to the detachment and disorganization of microtubules constituting the irreversible activation. In the case of weak stimulus, platelet trajectory reverses before the area III, following the return of intracellular calcium concentration to the baseline. This simple physical framework for revesible/irreversible activation may shed light on the platelet decision-making mechanisms [41]. However, the relationship between the intracellular calcium concentration and the overcurvature of marginal band is an important topic for future research. Our model of platelet activation is animated in the S1 Movie including the stages of marginal band bending and cell spherizing, after which pseudopodia extrusion can be additionally simulated (please see below for detalis). In this video the cell volume is constant.

## Discussion

### Mechanisms of marginal band coiling

The marginal band coiling implies the action of forces. Mechanical work of these forces is stored as a bending energy of microtubules in a bundle, which is proportional to the local squared curvature. As a natural first approximation, we assume that the amount of stored energy per unit length should not vary significantly, which implies nearly constant curvature of the coiled marginal band. However, only detailed considerations of possible mechanisms can prove this hypothesis.

The platelet cytoskeleton comprises the following major elements: marginal band of microtubules, submembrane cortex, actin filaments and the molecular motors associated with microtubules. It is widely accepted to attribute marginal band coiling solely to the influence of cortical tension. However, platelet activation affects every cytoskeletal subsystem in various ways, and each of them can contribute to the shape change:

- The increase of Ca^2+^ concentration shifts the balance of antagonistic molecular motors dynein and kinesin [42,43], which causes relative sliding of microtubules in the marginal band. This may lead to the internal stress in the bundle and hence the excessive curvature.
- The same motors, anchored outside the marginal band, could force it to elongate and rest against the submembrane cortex [28].
- Ca^2+^ may directly cause the depolymerization of tubulin [44,45] and thus affect the rigidity of the microtubules ring. Early studies revealed the transient partial depolymerization of tubulin during shape change [46,47]. Microtubules disassembly is also relevant for cold-induced shape change [48,49].
- Calcium-independent, Rho-kinase mediated pathway contribute to the maintenance of rounded cell shape, probably also by the depolymerization of tubulin [7].
- Ca^2+^ directly increases cortical tension by initiating actin-myosin interaction [50]. Recently, Dmitrieff et al [35] performed a numerical simulations of marginal band coiling initiated by this mechanism and showed good agreement with experiment. In contrast to the present paper, the cell shape was described as an ellipsoid, and no analythical description of the marginal band was proposed.
- Ca^2+^ activates gelsolin, a molecule that severs actin filaments. Although the actin network is not the major factor maintaining the discoid platelet shape [26], it does contribute to disk-to-sphere transformation [51].

The roles of each mechanism are to be studied; however, all of them lead to approximately the same morphology of the marginal band, which we discuss in detail in the next section.

### Mechanisms leading to the excessive curvature

**Fig 3:**
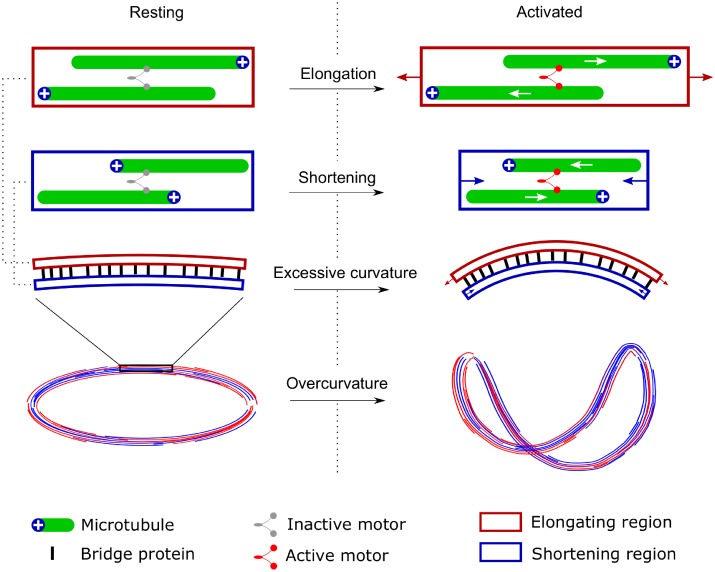
Possible mechanism of the overcurvature formation. Molecular motors induce relative sliding of microtubules, which are additionally cross-linked by bridge proteins. This leads to the formation of excessive curvature in the microtubules bundle, which manifests itself by the out-of-plane coiling of marginal band.

The concept of the excessive curvature naturally arises in the first mentioned mechanism. In platelets, the marginal band consists of multiple microtubules with different polarity [52]. Molecular motors exert sliding forces on the microtubules towards the plus-end (in case of dynein). Two microtubules with opposite polarity may be joined by dynein [53], which is inactive (or counterbalanced by kinesin) in the resting state. Activation shifts the motor balance [28], which leads to microtubule sliding and formation of elongating and shortening regions in the marginal band (Fig 3).

Additionally, these regions are cross-linked by protein bridges [35]. This resembles the mechanism of flagellar beating, also driven by dynein-mediated sliding of cross-linked microtubules, which leads to constant-curvature bending [54]. Another analogy is the bimetallic strip, which bends during heating due to the different thermal expansions of its joined metallic constituents. If the initial structure is the closed planar ring, formation of excessive curvature induces out-of-plane coiling. Experiment of Mouthuy et al [29] indeed showed that bimetallic ring forms the overcurved circle after temperature change.

The described interplay between microtubules and molecular motors in the marginal band may constitute the major or at least significant factor of its coiling in platelets. Nevertheless, different mechanisms of the excessive curvature formation are possible. For instance, dyneins or kinesins can be anchored in the submembrane cortex. Cortex influence on the microtubules bundle are discussed in the following section.

### Deviations of marginl band from the overcurved ring

The influence of cell surface tension, which is implied in the majority of above-mentioned mechanisms, may lead to the coiling of marginal band with non-constant curvature. Direct simulation of filamentous ring growth in flexible confinements shows distinct packing patterns depending upon elastic properties of the filament and the shell [55]. However, in each case the ring coils to a saddle-like structure at the initial step, very similar to the marginal band coiling in platelets. It is not clear whether these structures obey the constant curvature condition or the similarity is only visual. From physical point of view, in the limit of large surface tension the shell would adopt spherical shape, irrespectively to the internal cytoskeletal elements. It is useful to consider the case when microtubules lie on spherical surface, at the same time minimizing the bending energy. The solution of the corresponding mathematical problem was obtained by Guven et al [56]. The only stable configuration possesses the same *D*_2d_ symmetry as an overcurved circles used in the present paper. However, while the normal component of its curvature vector is constant, geodesic component oscillates around zero with non-negligible amplitude. The total curvature can be found from these two components by the Pythagorean theorem; it would also be the sum of some constant value and oscillations (generally non-symmetrical). For this point, we conclude that the overcurved circle is suitable as the first approximation in the limit of large surface tension, as well as in other discussed cases. It allows us to capture symmetry relations and the overall structure of platelets shapes. However, for the studies which rely on platelets microstructural properties our method may not be accurate enough. For instance, Zhang et al [57] had to directly model the molecular-scale intra-platelet constituents to account for the influence of hemodynamic stress on the cells (i.e., shear-induced activation). Another experimental study [24] deals with platelet deformability at nanoscale level and the ability to exert contraction force. This force was successfully used for the targeted drug delivery using platelet-hybridized capsules, which rupture upon activation [58]. Generally, in such constraint conditions, one could expect the marginal band to deform and adopt complex shape, dependent on the applied force. It is an interesting research direction to consider perturbations of overcurved circle, such as curvature oscillations and unidirectional compression.

## Methods

### Simulation of platelet marginal band

We model the marginal band of platelets as an overcurved ring (tube) with circular cross-section. The central line consists of 4 lobes, each of them being a part of the Salkowski curve *γ*_m_(*t*) = (*x*_m_(*t*), *y*_m_(*t*), *z*_m_(*t*)) [59], where *t* is a parameter and the internal variable *m* can be uniquely (numerically) determined from overcurvature. The only modification is that we normalize the overcurved circle so that its length equals 2π for all values of *O*_p_, denoting the normalization coefficient as 1/*R*. Tubular surface is defined as follows: for each point on the *γ*_m_(*t*)/*R*, we take a vector along the curve normal with the length *r*/*R*, and rotate it around the tangential direction. End of the vector gives the circle, which would be the cross-section of the tube at this particular point. In the Frenet frame (tangent, normal, binormal) the vector is defined by

$$r=(0,\frac{r}{R}\text{cos}\varphi ,\frac{r}{R}\text{sin}\varphi )$$ (1)

where *φ* is the angle of rotation. Now we should transform the coordinates to the laboratory reference frame. For this purpose, one can use the transition matrix to the Frenet frame from [59] and its inverse. We obtain

$$\begin{array}{l} T={\left(\begin{array}{ccc} -\text{cos}\;\;t\text{cos}\;\;\;\text{cos}\;\;\;nt-n\text{sin}\;t\text{sin}\;nt & -\text{cos}\;\;nt\text{sin}\;t+n\;\text{cos}\;\;t\text{sin}\;nt & -\frac{n}{m}\text{sin}\;nt\\ \frac{n}{m}\text{sin}\;t & -\frac{n}{m}\text{cos}\;\;t & -1\\ n\text{cos}\;\;nt\;\text{sin}\;t-\text{cos}\;\;t\text{sin}\;nt & -n\text{cos}\;\;t\;\text{cos}\;\;nt-\text{sin}\;t\text{sin}\;nt & \frac{n}{m}\text{cos}\;\;t \end{array}\right)}^{-1}=\\ =\frac{1}{n(1+m^{2})}\left(\begin{array}{ccc} -(1+m^{2})n\text{cos}\;\;t\text{cos}\;\;nt-m^{2}\text{sin}\;t\text{sin}\;nt & m\text{sin}\;t & m^{2}\text{sin}\;t\text{cos}\;\;nt-n(1+m^{2})\text{cos}\;\;t\text{sin}\;nt\\ -(1+m^{2})n\text{cos}\;\;nt\text{sin}\;t+m^{2}\text{cos}\;\;t\text{sin}\;nt & -m\text{cos}\;\;t & m^{2}\text{cos}\;\;t\text{cos}\;\;nt-n(1+m^{2})\text{sin}\;t\text{sin}\;nt\\ -m\text{sin}\;nt & -m^{2} & m\text{cos}\;\;nt \end{array}\right), \end{array}$$ (2)

where $n=\frac{m}{\sqrt{1+m^{2}}}$. Finally, two-parametric expression of the tube surface (for one lobe) is as follows:

$$M(t,\varphi )=\frac{\gamma_{m}(t)}{R}+\frac{r}{R}(T_{12}\text{cos}\varphi +T_{13}\text{sin}\varphi ,T_{22}\text{cos}\varphi +T_{23}\text{sin}\varphi ,z_{m}(t)+T_{32}\text{cos}\varphi +T_{33}\text{sin}\varphi )$$ (3)

Four copies of (3) are then rotated, translated, and joined using the procedure described in [29] to form an overcurved ring with a circular cross-section.

### Cell surface of resting platelets

**Fig 4:**
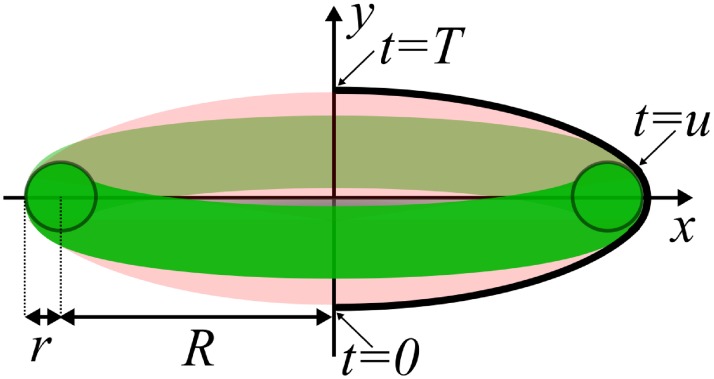
Illustration of platelet surface optimization problem. The surface is made by the revolution of profile {*x*(*t*), *y*(*t*)} (thick line) around the ordinate axis. It consists of two parts: free (light red) and attached to the marginal band (green). Point *t* = *u* corresponds to the contact between one of the mobile subdomain and the fixed part, and points *t* = 0 and *t* = *T* are where the the surface intersects the axis *x* = 0.

Inside resting platelets microtubules are organized in a circumferential ring, which, under our assumption of circular marginal band cross-section, corresponds to the torus. Its radii are *R* and *r* (Fig 4).

The surface adopts configuration with minimal area, while it must contain the torus and confine certain cellular volume *V*. This problem possesses axial symmetry, and we assume that the sought surface is also axisymmetric. So we further consider a sectional plane passing through the axis of revolution (defined to be the *y*-axis). Another coordinate axis in this plane is the *x*-axis, which belongs to the torus symmetry plane. Additionally we assume that the platelet membrane is a closed bounded surface with zero genus, i.e. has no holes, in accordance with all existing platelet images.

We seek for the parametric profile *x*(*t*) ≥ 0, *y*(*t*), *t* ∈ [0,*T*], for which the surface of revolution has minimal area:

$$S=\overset{T}{\underset{0}{\int }}2\pi x\sqrt{x\prime^{2}+y\prime^{2}}\text{d}t\to \text{min}$$ (4a)

subject to

$$V=\overset{T}{\underset{0}{\int }}\pi x^{2}y\prime \text{d}t=\text{const},$$ (4b)

$${\left(x-R\right)}^{2}+y^{2}\geq r^{2},$$ (4c)

x(0)=x(T)=0, (4d)

where the prime denotes differentiation over *t*, and condition (4d) follows from the zero-genus assumption above.

Condition (4c) makes the problem distinct from that of finding constant mean curvature surface with free boundary. However, generally the profile consists of two parts: one is attached to the marginal band and follows its exterior and the other is free (both of them may consist of several disjoint components). From physical considerations, the free part should obey the constant-mean-curvature condition, which (in the case of axial symmetry) leads to the family of Delaunay surfaces [60]. The only Delaunay surface which satisfies condition (4d) is a sphere, so one can conclude that free-surface parts intersecting the symmetry axis are necessarily spherical caps. Moreover, those caps have the same radius so that the whole free surface has the same mean curvature (including sign), which physically corresponds to constant pressure of the surface-tension forces. The mean curvature of the attached part equals that of the marginal band, which is always larger than that of the free part, since the latter spans the whole diameter of the torus. And it is the reaction force of the rigid marginal band which compensates for the excessive surface tenstion. If the free part would attach to only one side of the torus (when considered inside the section of Fig 4), then it would have even larger mean curvature than that of the marginal band; hence, such free parts are impossible.

In the following we obtain the same solution formally from the problem statement (4). By introducing the slack variable *f* ^2^ = (*x*−*R*)^2^ + *y*^2^ − *r*^2^, we transform condition (4c) into equality. Application of Euler-Lagrange multiplier theorem leads to the minimization of functional

$$\overset{b}{\underset{0}{\int }}L(t,x,x\prime ,y,y\prime ,f,f\prime ,\lambda_{2},\lambda_{2}^{\prime })\text{d}t,L=\text{2}\pi x\sqrt{x\prime^{2}+y\prime^{2}}-2\lambda_{1}\pi x^{2}y\prime -\lambda_{2}[(x-R^{2})+y^{2}-r^{2}-f^{2}],$$ (5)

where 2*λ*_1_ and *λ*_2_(*t*) are Lagrange multipliers (factor of 2 is introduced for simplicity). The set of equations follows:

$$\{\begin{array}{ll} 2\pi \sqrt{x\prime^{2}+y\prime^{2}}-4\pi \lambda_{1}xy\prime -2\lambda_{2}(x-R)-\frac{\text{d}}{\text{d}t}\left(\frac{2\pi xx\prime }{\sqrt{x\prime^{2}+y\prime^{2}}}\right)=0 & \left(6\text{a}\right)\\ -2\lambda_{2}y-\frac{\text{d}}{\text{d}t}\left(\frac{2\pi xy\prime }{\sqrt{x\prime^{2}+y\prime^{2}}}-2\lambda_{1}\pi x^{2}\right)=0 & \left(6\text{b}\right)\\ {\left(x-R\right)}^{2}+y^{2}-r^{2}-f^{2}=0 & \left(6\text{c}\right)\\ 2\lambda_{2}f=0 & \left(6\text{d}\right) \end{array}$$

Let us first consider the segments for which inequality (4b) is not active, i.e. when *f* ^2^(*t*) > 0. Then Eq (6d) gives *λ*_2_(*t*) = 0, and Eq (6b) yields:

$$\frac{xy\prime }{\sqrt{x\prime^{2}+y\prime^{2}}}-\lambda_{1}x^{2}=C,$$ (7)

where *C* is constant for each continuous segment (interval of *t*), but may differ between them. It can be shown that general solutions of Eq (7) are Delaunay surfaces with constant mean curvature. However, as discussed above, there can be at most two such free segments, containing *t* = 0 and *t* = *T*, respectively. Then condition (4d) implies *C* = 0 for all of these segments. Hence, Eq (6a) and (6b) are transformed into

$$\sqrt{x\prime^{2}+y\prime^{2}}-2\lambda_{1}xy\prime =\frac{\text{d}}{\text{d}t}\left(\frac{xx\prime }{\sqrt{x\prime^{2}+y\prime^{2}}}\right),$$ (8a)

$$y\prime =\lambda_{1}x\sqrt{x\prime^{2}+y\prime^{2}}.$$ (8b)

Eq (8b) implies

$$x^{2}+{\left(y-b\right)}^{2}=\lambda_{1}^{-2},\;\text{sgn}(y\prime )=\text{sgn}(\lambda_{1}),$$ (9)

which is a circle with the center on the *y*-axis and radius 1/|*λ*_1_|. Further, we additionally assume that our parametrization is counter clockwise, i.e. *y*(0) < *y*(*T*), then Eq (9) corresponds to a circle with signed outward curvature equal to *λ*_1_. Interestingly, Eq (8a) is automatically satisfied for any such solution. Considering two possible free segments, we note that integration constant *b* may differ, but *λ*_1_ is the same. The latter corresponds to mean curvature being constant over all free segments.

If the attached segment has zero length, then the profile is a single semicircle, and the whole platelet surface is a sphere containing the marginal band inside (potentially touching it). Otherwise we additionally need the boundary conditions between the attached and free segments, e.g. at the point *t = u*. Naturally, both *x*(*t*) and *y*(*t*) are continuous at *t* = *u*. Further, we apply Weierstrass–Erdmann conditions:

$$\begin{array}{cc} {\frac{\partial L}{\partial x\prime }|}_{t=u-0}^{t=u+0}=0 & \Rightarrow {\frac{x\prime }{\sqrt{x\prime^{2}+y\prime^{2}}}|}_{t=u-0}^{t=u+0}=0,\\ {\frac{\partial L}{\partial y\prime }|}_{t=u-0}^{t=u+0}=0 & \Rightarrow {\frac{y\prime }{\sqrt{x\prime^{2}+y\prime^{2}}}|}_{t=u-0}^{t=u+0}=0, \end{array}$$ (10)

noting that the rest of those conditions are trivial:

$$\begin{array}{l} \frac{\partial L}{\partial f\prime }=\frac{\partial L}{\partial \lambda_{2}^{\prime }}=0,\\ L-x\prime \frac{\partial L}{\partial x\prime }-y\prime \frac{\partial L}{\partial y\prime }-f\prime \frac{\partial L}{\partial f\prime }-\lambda_{2}^{\prime }\frac{\partial L}{\partial \lambda_{2}^{\prime }}=-\lambda_{2}[{\left(x-R\right)}^{2}+y^{2}-r^{2}-f^{2}]=0, \end{array}$$ (11)

where the last equality is due to Eq (6c). While Eq (10) does not guarantee continuity of *x*′ and *y*′ at *t* = *u*, it implies the continuity of tangent direction (i.e. *x*′/*y*′ or *y*′/*x*′) at *t* = *u*. Physically, it is also justified if there are no adhesion forces (at least near the contact line) between the marginal band and the cell membrane. In this case only tension forces are present, which are directed along each surface segment. For these forces to cancel out, they must be antiparallel, so the segments should join smoothly. The equivivalent boundary condition is the zero contact angle between the cell surface and the marginal band (i.e., “superhydrophilic” [61] marginal band exterior). Note that this physical interpretation remains valid in the general case of activated platelets (no axial symmetry).

In the case of resting platelets, the condition (10) together with *f* = 0 and Eq (9) implies that the marginal-band circle is tangent to the semicircle of Eq (9), dividing the latter into two parts, one of which belongs to the sought profile. The centers of two circles and contact point lie on a line, which leads to the following equation for *a* = *x*(*u*):

$$\lambda_{1}a=\frac{a-R}{r},\;\left|a-R\right|\leq r,$$ (12)

for which *y*(*u*) can take one of the two values with opposite signs. Note that positive and negative signs of *λ*_1_ corresponds to internal and external tangency, respectively. The solution of Eq (12) is given by:

$$a=\frac{R}{1-\lambda_{1}r},\;-\frac{1}{R-r}\leq \lambda_{1}\leq \frac{1}{R+r}.$$ (13)

If the radius of free-segment circle is smaller than the torus hole radius *R* − *r* (|*λ*_1_| is too large), joining of two curves is not possible at all. While a small semicircle can minimize the functional (5), it does not surround the marginal band. For larger free-segment radius but negative *λ*_1_, there are two circles externally tangent to the marginal band. There are two ways to combine the parts of these circles with parts of the marginal band into a smooth profile, but only one of them, corresponding to a biconcave disk, surrounds the marginal band. Similarly, for positive *λ*_1_ there are two cirles, to which the marginal band is internally tangent. In principle, the segments of these circles can be combined smoothly with the marginal band in three ways, but two of such profiles would only touch the marginal band at a single contact point corresponding to the semicircle solutions described above. The remaining solution is a biconvex one. Importantly, all solutions except a semicircle are symmetric with respect to the *x*-axis and are uniquely determined by the value of *λ*_1_ [cf. Eq (13)].

The only additional complication is that the segments of the biconcave shape must not intersect, i.e. each concave segment should not cross the *x*-axis (zero-genus assumption). The minimum possible curvature is thus −2*r*/(*R*^2^ –*r*^2^). For smaller (more negative) curvatures there exist surfaces with flat double layer in the center, that potentially may be obtained for real platelets by removing sufficient amount of volume. Still, we do not consider them here, as they require modification of the functional (5) to explicitly include the double-layer part. Finally, all symmetric solutions can be expressed with the following formula:

$$y(x)=\pm \left\{\begin{array}{ll} \frac{1}{\lambda_{1}}\left(\sqrt{1-{\left(\lambda_{1}x\right)}^{2}}-\sqrt{{\left(1-\lambda_{1}r\right)}^{2}-{\left(\lambda_{1}R\right)}^{2}}\right), & 0\leq x\leq \frac{R}{1-\lambda_{1}r}\\ \sqrt{r^{2}-{\left(x-R\right)}^{2}}, & \frac{R}{1-\lambda_{1}r}<x\leq R+r \end{array}\right\},-\frac{2r}{R^{2}-r^{2}}\leq \lambda_{1}\leq \frac{1}{R+r}$$ (14)

It can be shown that *y*(*x*) is smooth with respect to *λ*_1_ in the whole specified range, including *λ*_1_ = 0. It is straightforward to obtain the cell volume *V* from Eq (13), but the final expression is rather cumbersome. Importantly, *V* monotonically increases with *λ*_1_, which also follows from not-crossing of profiles for different *λ*_1_, i.e. for any two profiles, one completely surrounds the other (potentially touching it).

**Fig 5:**
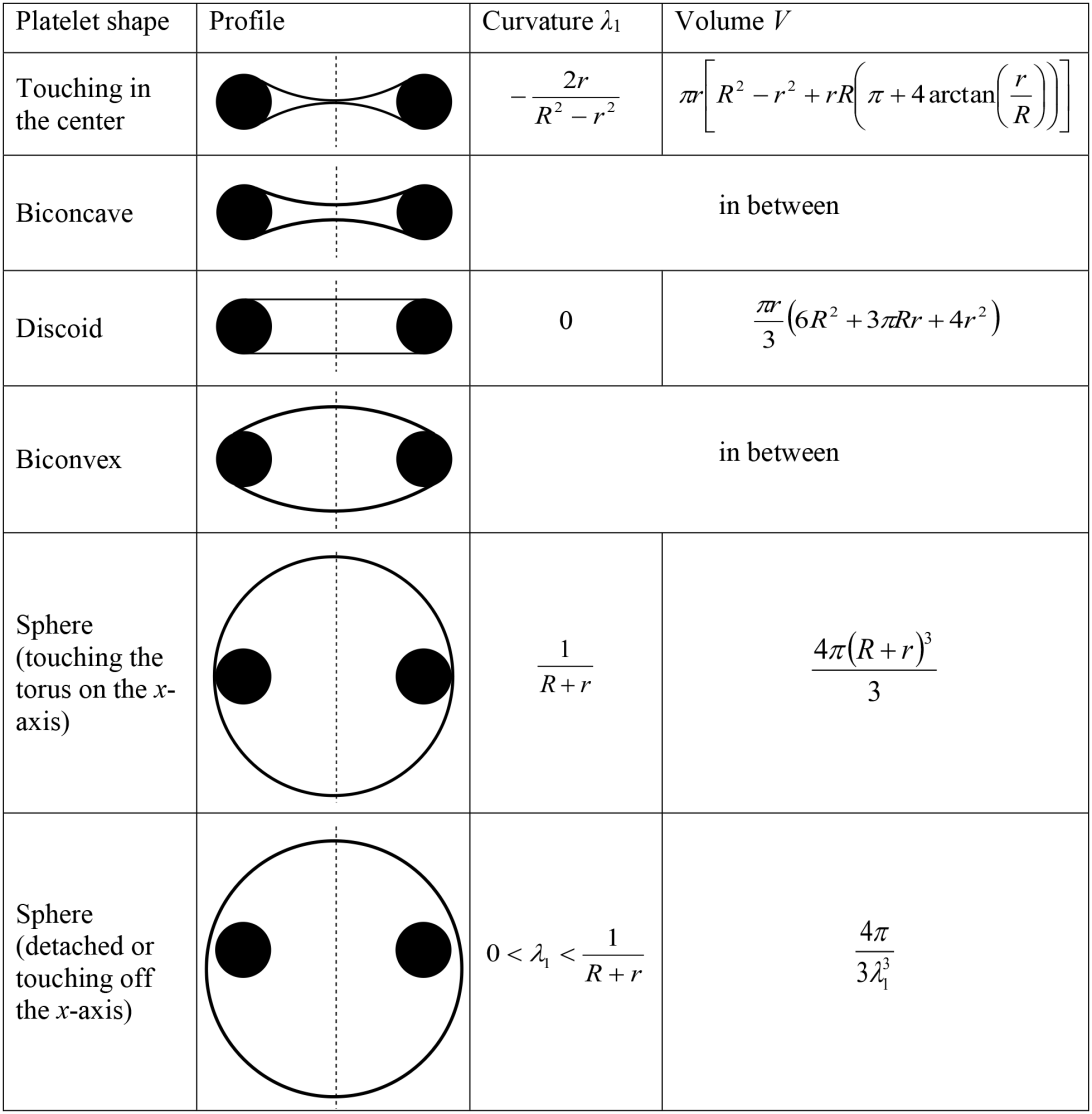
All possible axisymmetric profiles of resting platelets as obtained by the solution of variational problem (4). Black circles correspond to the marginal band cross-sections. Volume increases from top to bottom, curvature increases when going from the first to the fifth row, but decreases for the last row.

We summarize all the obtained morphologies of resting platelets, and provide the characteristic values of *λ*_1_ and *V* in a table presented in Fig 5.

Volume increases from top to bottom and *λ*_1_ is a single-valued function of *V* for all ranges of shapes. Thus, the variational problem (4) has unique solution (except for vertical translations of a semicircle profiles). If only symmetric attached profiles are considered (bounded *V*), curvature *λ*_1_ is a convenient internal parameter, bijectively related to *V*.

### Cell surface in case of coiled marginal band

**Fig 6:**
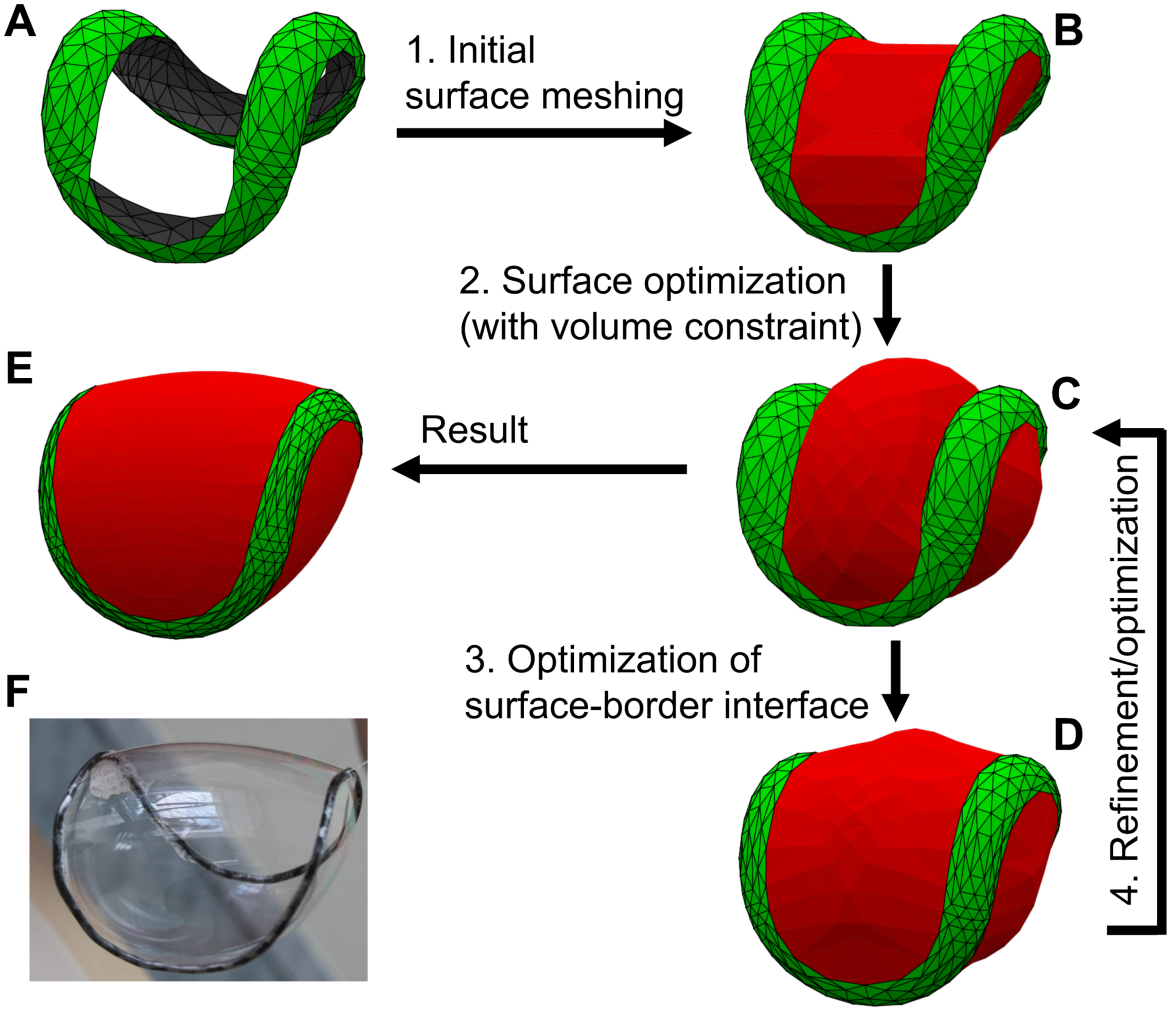
Scheme of the construction of blood platelet shape model (A–E) and the soap bubble supported by the overcurved wire ring (F), which presents the real-world approximation of the optimization problem solution for the mobile surface parts.

The numerical optimization algorithm of platelets shape for arbitrary overcurvature of the marginal band was briefly described above and summarized in Fig 6.

It is implemented as the Surface Evolver [62] script, available as S1 File. If cell parameters correspond to non-sphered shape (volume is not too large; cf. Fig 5), the optimization procedure begins. At the first step we construct the surface of overcurved ring (3) from given *O*_p_ and *r/R* with *φ* varying from *φ*_d_ to 2π−*φ*_d_. For this purpose, we define facets that are constrained to lie on boundary and refine them several times using intrinsic Surface Evolver procedure (r command). The obtained surface (Fig 5A) simulates the region where cell membrane sticks to the marginal band. It is excluded from the following optimization (fixed). We also define two mobile parts of the surface: first is bounded by the space curve *φ* = *φ*_d_ and the second by *φ* = 2π−*φ*_d_, corresponding to the upper and lower cell halves (Fig 5B). These parts are refined and optimized to have minimal area with the requirement that the overall surface should confine the prescribed volume *V*. Standard procedure (g and r commands) is used in this step.

Next, we optimize the interface at the border between mobile and fixed parts. The aim is to make the surface smooth, i.e., minimize the contact angle, analogously to the condition (10) in the axisymmetric case. So we attenuate the contact position *φ*_c_(*t*) along the border. For this purpose we implemented special procedure bopt, which computes the new positions of vertices on the overcurved circle (simple one-step gradient optimization). Next the surface is optimized, which again leads to the non-zero contact angle. After several iterations, we obtain reasonably converged shape. The typical process is shown in S2 Movie. We stress that the physical reasoning behind our method requires both contact angles and the free-surface to be simultaneously optimized (as is in Fig 5E). The two-level iterative optimization is just one of the possible options to achieve this goal. Note also that we do not consider the influence of the membrane tension on the marginal band. Instead, we assume that it is an overcurved ring with already known shape.

**Fig 7:**
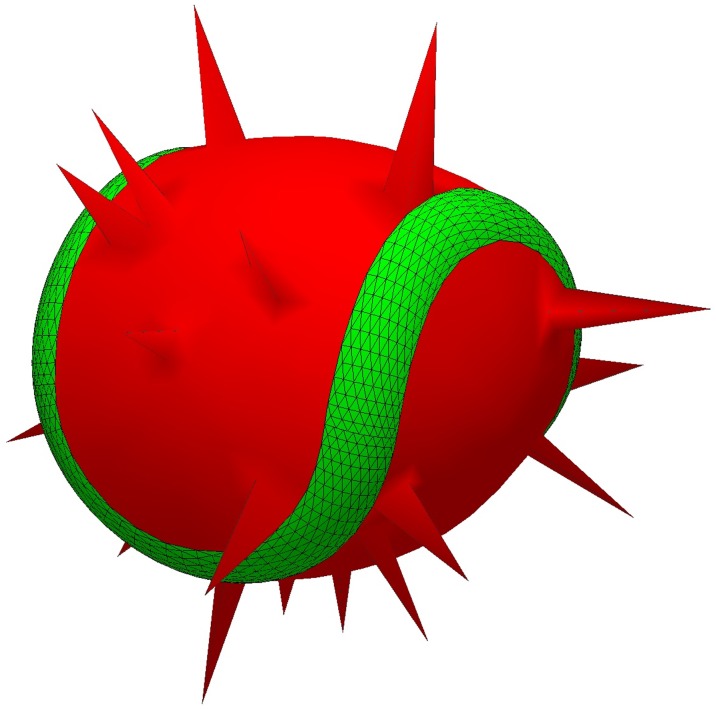
Example of platelet model with 20 pseudopodia. Their total relative volume is 0.01.

Characteristic feature of the activated platelets is the presence of pseudopodia, which influence the physical properties of cells. For instance, they can increase the hydrodynamic radius of cells. Light-scattering patterns also differ for platelet models with or without pseudopodia [19]. However, the main effect in this case is the decrease of volume of the central cell body, because protrusions with thickness less than the wavelength are weak scatterers. Anyway, we implemented the possibility to add simple membrane protrusions (resembling pseudopodia) by the following procedure. First, we fix the obtained surface except several vertices, randomly selected on the mobile surface parts. Next, we increase the target cell volume by a small amount, multiplying it by (1+ ppv), where ppv is a user-controlled parameter representing the relative volume of pseudopodia. On the next step, we perform a single evolver iteration (g), which forces the unfixed vertices to shoot out (Fig 7).

It should be stressed that this is a zero-order approximation of pseudopodia, mainly to give the shape model better visual resemblance to fully-activated platelet. It may correspond physically to the extrusion of feeble sites of platelet surface (i.e., membrane loci where no or little filaments are attached) by an intracellular pressure, but we do not consider microtubules or actin polymerization, which are the main mechanistical feature of the process.

### Example of application: Light scattering simulations

**Fig 8:**
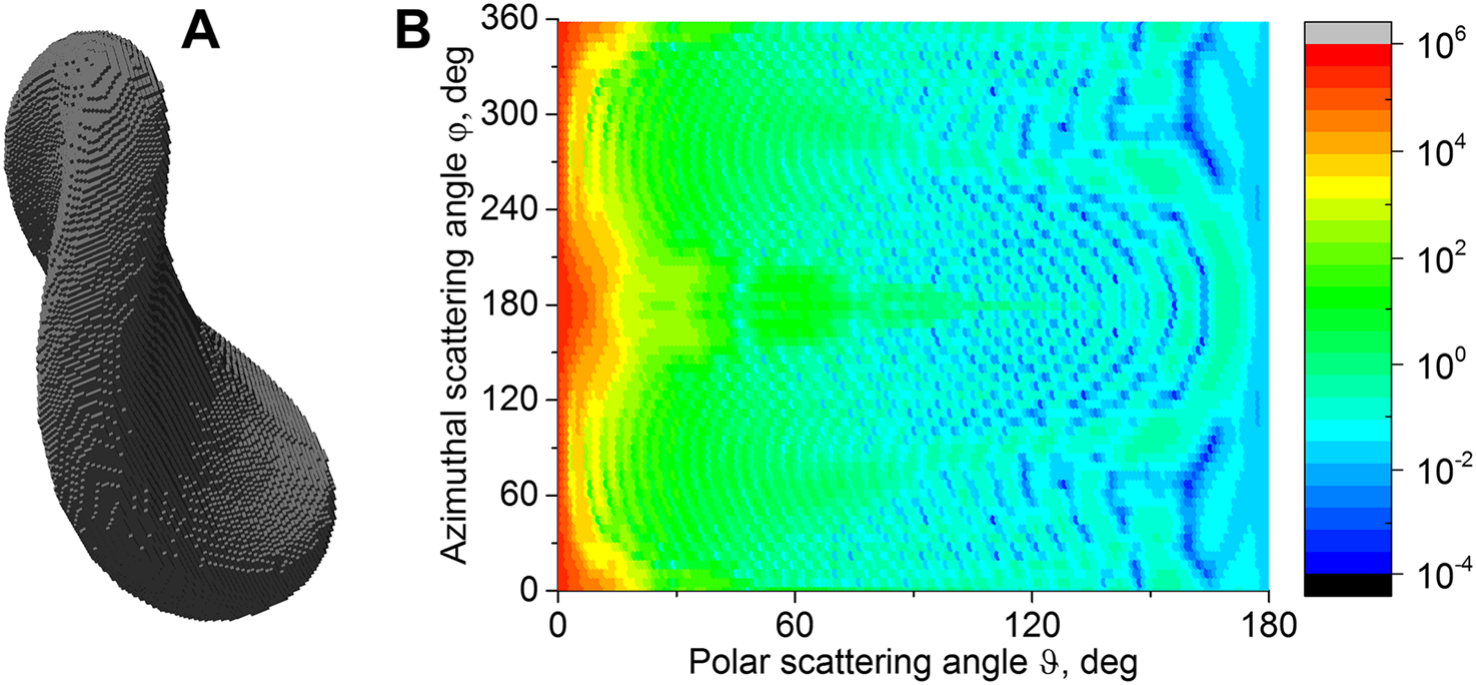
A. Light scattering model obtained by filling of platelet model with dipoles; incident wave propagates from below. B. Result of light scattering simulation—controur plot of *S*_11_ element of the Mueller matrix in logarithmic scale versus polar and azimuthal scattering angles.

In order to demonstrate the relevance of the proposed shape model for platelets research, we performed simulation of light scattering by these shapes using the discrete-dipole approximation (DDA). These simulations can be implemented in single script using the surface evolver’s ability to run system commands. The workflow consists of:

1. Simulation of 3D platelet shape and saving it as an .obj file, which is accomplished by our script for the Surface Evolver. The .obj format is acceptable by a wide variety of software for 3D modeling and computations. Basically, it contains the surface mesh.
2. Filling the shape interior with dipoles using Point in Polyhedron (PIP) tool [63]. The DDA involves volume-integral method for the solution of Maxwell equations, so volume discretization of the particle is required [64]. This step currently is a computational bottleneck since each volume element (dipole) of a regular grid is tested independently for being inside the platelet surface. Hence, the time is proportional to both the number of dipoles and the number of surface mesh elements. The only parameter for PIP is the number of dipoles per cell dimension along the *x*-axis. To evaluate this parameter, we employ the standard DDA rule of thumb of 10 dipoles per the wavelength [65]. The discretization takes 5–10 min on a single processor core. An example of obtained dipoles configuration is shown in Fig 8A.
3. Simulation of light scattering using ADDA code v.1.2 [65]. ADDA is open-source software for discrete-dipole approximation. It uses Fast Fourier Transform for acceleration of vector-matrix multiplication, so that light scattering simulation time is nearly proportional to the number of dipoles. In case of platelets, the simulation takes around 1–2 minutes. The scatterer is defined by the shape (obtained in the previous step), size parameter, refractive index, and orientation.

We performed simulation for the particle oriented as shown in Fig 8A, where the incident wave propagates from below. The refractive index of the platelet model was set to 1.03 (relative to the medium), the volume to 11.5 fL, and the wavelength in vacuum—to 405 nm, while medium refractive index was 1.337. In Fig 8B, the intensity of scattered light (more precisely, the Mueller matrix element *S*_11_) is shown as a function of polar and azimuthal scattering angles. The distribution of intensity shows complex features and is distinctive from smooth pattern of oblate spheroid described in [66]. Note also that the presented model of activated platelet not only possess one or two additional morphological parameter in comparison with models of resting platelets, but also lacks axial symmetry, which brings about the influence of Euler angle *γ* on the light-scattering patterns. These three additional parameters complicate the solution of the inverse light scattering problem and hinder the incorporation of the presented model into the existing algorithms [9,10]. Further theoretical and experimental research concerning optical properties of blood platelets is needed to extract new information and develop novel diagnostic techniques.

## Supporting information

S1 Movie Simulation of platelet activation. The simulated platelet shape is shown as the overcurvature increases from 1 to 2.3. The relative cell volume remains constant (2.0), as well as the relative thickness of marginal band (0.2). After overcurvature of 2.26, the cell becames spherical, as the convoluted marginal band fits inside.
(AVI)

S2 Movie Surface optimization. The surface optimization for platelet model is shown in real-time for overcurvature 1.8, relative cell volume 1.8 and relative marginal band thickness 0.2.
(AVI)

S1 File Script for simulation of platelet shape from given parameters: Overcurvature *O*_p_, relative volume *v* and dimensionless radius of marginal band cross-section *r*/*R*. Two additional parameters may be set: the relative volume of pseudopodia and .obj file where to save the optimized surface. All parameters are set at first lines of the script as follows:
parameter OC ≔ 1.8  //Overcurvature
parameter Vol ≔ 1.8  //Relative volume, V/R^3
parameter Rad ≔ 0.2  //Relative half-thickness of the marginal band, r/R
parameter ppv ≔ 0.0  //Relative volume of pseudopodia; 0 if not needed
parameter sobj ≔ ""  //Filename to save .obj file; blank if not needed
The Surface Evolver (http://facstaff.susqu.edu/brakke/evolver/evolver.html) is needed to run the script. Once it is installed, the script can be launched by double click or from command line. Optimization is displayed in standard surface evolver interactive 3D window.
The steps of optimization procedure are defined in the bottom of the file. By default, the implemented script gives reasonable results for all tested parameters. However, the procedure converges faster for models with smaller volumes, especially with concave surface. So, for some values of parameters, the number of iterations can be significantly reduced.
(FE) Click here for additional data file.
